# Supplementary material for: Seminoma and Embryonal Carcinoma Footprints Identified by Analysis of Integrated Genome-Wide Epigenetic and Expression Profiles of Germ Cell Cancer Cell Lines
Source: PLoS One. 2014 Jun 2;9(6):e98330. doi: 10.1371/journal.pone.0098330 (PMC4041891; doi:10.1371/journal.pone.0098330)
Supplement: File S1 — ZIP file containing DMRforPairs output for significant regions. Please start from the html files. (ZIP) [file pone.0098330.s008.zip › significant.html]

DMRforPairs output generated on Sun Oct 20 16:11:16 2013. Identified regions were set to contain at least 4 probes with a maximum distance of 200 bp between individual probes (n=30306). Regions in which median methylation levels (M-values) between the samples differed at least |1.4| (n=5139) (=relevant) were tested for statistical significance (significant: p<0.05; multiple testing adjusted, n=143). The folowing samples were studied: TCam2,NCCIT. Chromosomal positions are indicated in bp. N indicates the number of probes in a region. ID indicates the region ID. Values in the columns per sample indicate median beta values. dM indicates the largest median difference (absolute) between any of the sample pairs. p indicates uncorrected p-value from Mann-Whitney U test (n=2) or Kruskal Wallis test (n>2). p.adj denotes the multiple testing corrected p-value (method:bonferroni). Gene symbols of overlapping transcripts are listed for the exact region and within a margin of 10000 bp of the region.

| Thumbnail | Chr | Start | End | Links | Length | n | ID | Class | TCam2 | NCCIT | Gene.Symbol | dM | p | p.adj |
| --- | --- | --- | --- | --- | --- | --- | --- | --- | --- | --- | --- | --- | --- | --- |
|  | 4 | 144480206 | 144480561 | PDFSTATSENSEMBLUCSC | 356 | 11 | 30566 | tss | 0.00812 | 0.8074 | SMARCA5; GUSBP5 (margin: SMARCA5; GUSBP5; RP11-481K16.2) | 10.03 | 2.84e-06 | 1.46e-02 |
|  | 4 | 144480206 | 144480740 | PDFSTATSENSEMBLUCSC | 535 | 12 | 31283 | island | 0.00838 | 0.8508 | SMARCA5; GUSBP5 (margin: SMARCA5; GUSBP5; RP11-481K16.2) | 9.83 | 7.40e-07 | 3.80e-03 |
|  | 4 | 103940711 | 103941300 | PDFSTATSENSEMBLUCSC | 590 | 11 | 31159 | island | 0.01663 | 0.8913 | SLC9B1; SLC9B2 (margin: SLC9B1; SLC9B2) | 9.62 | 2.84e-06 | 1.46e-02 |
|  | 7 | 95025611 | 95026248 | PDFSTATSENSEMBLUCSC | 638 | 20 | 38614 | island | 0.02405 | 0.9370 | PON1; PON3 (margin: PON1; PON3; PON2) | 9.60 | 1.45e-11 | 7.46e-08 |
|  | 1 | 161008127 | 161008977 | PDFSTATSENSEMBLUCSC | 851 | 11 | 3884 | island | 0.03083 | 0.9612 | F11R; TSTD1; USF1 (margin: RP11-544M22.3; F11R; TSTD1; USF1; ARHGAP30) | 9.59 | 2.84e-06 | 1.46e-02 |
|  | 7 | 95025736 | 95026248 | PDFSTATSENSEMBLUCSC | 513 | 19 | 37661 | tss | 0.02143 | 0.9363 | PON1; PON3 (margin: PON1; PON3; PON2) | 9.50 | 5.66e-11 | 2.91e-07 |
|  | 1 | 165737775 | 165738563 | PDFSTATSENSEMBLUCSC | 789 | 14 | 3921 | island | 0.89099 | 0.0154 | RP11-466F5.8; TMCO1 (margin: RP11-466F5.8; TMCO1) | 9.32 | 4.99e-08 | 2.56e-04 |
|  | 14 | 65006688 | 65007512 | PDFSTATSENSEMBLUCSC | 825 | 18 | 12907 | island | 0.03705 | 0.9017 | HSPA2; RP11-973N13.4 (margin: ZBTB1; HSPA2; PPP1R36; RP11-973N13.4; RP11-973N13.5; RP11-973N13.3) | 9.07 | 2.20e-10 | 1.13e-06 |
|  | 19 | 54290737 | 54291194 | PDFSTATSENSEMBLUCSC | 458 | 11 | 21003 | tss | 0.02519 | 0.9322 | MIR371A; MIR372; MIR373; AC008753.4; MIR371B (margin: AC008753.3; MIR371A; MIR372; MIR373; AC008753.4; MIR371B; NLRP12) | 8.99 | 2.84e-06 | 1.46e-02 |
|  | 14 | 65006688 | 65007171 | PDFSTATSENSEMBLUCSC | 484 | 15 | 12431 | tss | 0.04363 | 0.9014 | HSPA2; RP11-973N13.4 (margin: ZBTB1; HSPA2; PPP1R36; RP11-973N13.4; RP11-973N13.5) | 8.94 | 1.29e-08 | 6.63e-05 |
|  | 6 | 27832728 | 27833555 | PDFSTATSENSEMBLUCSC | 828 | 13 | 35507 | island | 0.02640 | 0.9417 | HIST1H2AL; HIST1H2BPS2; HIST1H1B (margin: HIST1H2BN; HIST1H2AL; HIST1H2BPS2; HIST1H1B; HIST1H3I; HIST1H4L) | 8.90 | 3.85e-07 | 1.98e-03 |
|  | 15 | 40544855 | 40545511 | PDFSTATSENSEMBLUCSC | 657 | 11 | 13261 | gene;island | 0.01977 | 0.8970 | RP11-133K1.2; PAK6; C15orf56 (margin: RP11-133K1.2; PAK6; C15orf56) | 8.73 | 2.84e-06 | 1.46e-02 |
|  | 10 | 134599783 | 134600998 | PDFSTATSENSEMBLUCSC | 1216 | 30 | 5432 | tss;island | 0.03311 | 0.9343 | RP11-288G11.3; NKX6-2 (margin: INPP5A; RP11-288G11.3; NKX6-2) | 8.67 | 1.69e-17 | 8.69e-14 |
|  | 6 | 26033394 | 26034145 | PDFSTATSENSEMBLUCSC | 752 | 13 | 34618 | tss;island | 0.01093 | 0.8134 | HIST1H3B; HIST1H2AB (margin: HIST1H4A; U91328.2; HIST1H3C; HIST1H4B; HIST1H3B; HIST1H2AB; HIST1H2BB) | 8.15 | 1.92e-07 | 9.88e-04 |
|  | 2 | 187713554 | 187714279 | PDFSTATSENSEMBLUCSC | 726 | 12 | 24912 | island | 0.03341 | 0.9143 | ZSWIM2 (margin: ZSWIM2) | 8.10 | 7.40e-07 | 3.80e-03 |
|  | 17 | 17109570 | 17110353 | PDFSTATSENSEMBLUCSC | 784 | 14 | 18027 | island | 0.05499 | 0.9432 | MPRIP; PLD6; RP11-45M22.4 (margin: MPRIP; PLD6; RP11-45M22.4; FLCN) | 7.98 | 9.97e-08 | 5.12e-04 |
|  | 13 | 36871646 | 36872346 | PDFSTATSENSEMBLUCSC | 701 | 14 | 11639 | island | 0.94549 | 0.0921 | SOHLH2; CCDC169-SOHLH2; CCDC169 (margin: SOHLH2; CCDC169-SOHLH2; CCDC169; SPG20) | 7.75 | 4.99e-08 | 2.56e-04 |
|  | 4 | 188916496 | 188916943 | PDFSTATSENSEMBLUCSC | 448 | 11 | 31437 | island | 0.96312 | 0.1185 | ZFP42 (margin: ZFP42) | 7.69 | 2.84e-06 | 1.46e-02 |
|  | 2 | 113341711 | 113342262 | PDFSTATSENSEMBLUCSC | 552 | 12 | 24586 | island | 0.02745 | 0.8502 | CHCHD5 (margin: POLR1B; CHCHD5; AC012442.5; AC012442.6; Y\_RNA) | 7.61 | 7.40e-07 | 3.80e-03 |
|  | 7 | 27204663 | 27205658 | PDFSTATSENSEMBLUCSC | 996 | 17 | 38292 | island | 0.02568 | 0.7755 | HOXA9; RP1-170O19.20 (margin: HOXA-AS3; RP1-170O19.21; HOXA-AS4; HOXA7; HOXA9; RP1-170O19.20; MIR196B; HOXA10) | 7.46 | 8.57e-10 | 4.40e-06 |
|  | 4 | 89618324 | 89619573 | PDFSTATSENSEMBLUCSC | 1250 | 20 | 30009 | gene | 0.04982 | 0.9162 | HERC3; NAP1L5 (margin: HERC3; FAM13A-AS1; NAP1L5) | 7.14 | 2.32e-08 | 1.19e-04 |
|  | 17 | 17109651 | 17110353 | PDFSTATSENSEMBLUCSC | 703 | 12 | 17129 | tss | 0.11191 | 0.9432 | MPRIP; PLD6; RP11-45M22.4 (margin: MPRIP; PLD6; RP11-45M22.4; FLCN) | 7.07 | 1.48e-06 | 7.60e-03 |
|  | 6 | 30698584 | 30698987 | PDFSTATSENSEMBLUCSC | 404 | 11 | 33755 | gene | 0.04332 | 0.8759 | FLOT1 (margin: TUBB; XXbac-BPG252P9.10; XXbac-BPG252P9.9; FLOT1; Y\_RNA; IER3) | 6.94 | 2.84e-06 | 1.46e-02 |
|  | 19 | 52391078 | 52391789 | PDFSTATSENSEMBLUCSC | 712 | 13 | 22309 | island | 0.17681 | 0.9579 | CTC-429C10.2; ZNF577; ZNF649 (margin: CTC-429C10.2; ZNF577; ZNF649) | 6.90 | 1.92e-07 | 9.88e-04 |
|  | 19 | 15342749 | 15343425 | PDFSTATSENSEMBLUCSC | 677 | 11 | 19867 | gene;island | 0.75907 | 0.0466 | EPHX3 (margin: AC004257.1; EPHX3; AC004257.3; BRD4) | 6.76 | 2.84e-06 | 1.46e-02 |
|  | 19 | 52900882 | 52901307 | PDFSTATSENSEMBLUCSC | 426 | 11 | 22322 | island | 0.02292 | 0.8385 | ZNF528; CTD-3018O17.3 (margin: ZNF880; ZNF528; CTD-3018O17.3) | 6.73 | 2.84e-06 | 1.46e-02 |
|  | 12 | 3862221 | 3862597 | PDFSTATSENSEMBLUCSC | 377 | 12 | 10140 | island | 0.04487 | 0.8103 | EFCAB4B (margin: EFCAB4B) | 6.73 | 7.40e-07 | 3.80e-03 |
|  | 6 | 79576851 | 79577260 | PDFSTATSENSEMBLUCSC | 410 | 11 | 36209 | island | 0.04090 | 0.7106 | IRAK1BP1 (margin: IRAK1BP1) | 6.70 | 2.84e-06 | 1.46e-02 |
|  | 19 | 12075601 | 12076348 | PDFSTATSENSEMBLUCSC | 748 | 11 | 21552 | island | 0.03051 | 0.6733 | ZNF763 (margin: ZNF763; CTD-2006C1.12) | 6.63 | 2.84e-06 | 1.46e-02 |
|  | X | 119005413 | 119006122 | PDFSTATSENSEMBLUCSC | 710 | 14 | 41095 | gene;tss;island | 0.01651 | 0.6247 | NDUFA1; RNF113A (margin: NDUFA1; RNF113A) | 6.62 | 4.99e-08 | 2.56e-04 |
|  | 14 | 75593334 | 75594058 | PDFSTATSENSEMBLUCSC | 725 | 14 | 12984 | island | 0.69509 | 0.0242 | RP11-950C14.7; NEK9 (margin: RP11-950C14.7; NEK9; HIF1AP1; TMED10) | 6.55 | 4.99e-08 | 2.56e-04 |
|  | 1 | 209979111 | 209979779 | PDFSTATSENSEMBLUCSC | 669 | 13 | 4154 | island | 0.02320 | 0.6680 | IRF6 (margin: IRF6) | 6.49 | 1.92e-07 | 9.88e-04 |
|  | 7 | 99517136 | 99517509 | PDFSTATSENSEMBLUCSC | 374 | 12 | 38677 | island | 0.80869 | 0.0329 | TRIM4 (margin: RP4-604G5.1; TRIM4; GJC3) | 6.49 | 7.40e-07 | 3.80e-03 |
|  | 20 | 61446962 | 61448325 | PDFSTATSENSEMBLUCSC | 1364 | 42 | 25843 | tss;island | 0.04147 | 0.7428 | OGFR; COL9A3 (margin: OGFR; COL9A3; OGFR-AS1) | 6.45 | 1.19e-24 | 6.12e-21 |
|  | 10 | 93392644 | 93393509 | PDFSTATSENSEMBLUCSC | 866 | 14 | 5913 | island | 0.03365 | 0.7551 | PPP1R3C (margin: PPP1R3C) | 6.41 | 4.99e-08 | 2.56e-04 |
|  | 10 | 49812686 | 49813434 | PDFSTATSENSEMBLUCSC | 749 | 13 | 5708 | island | 0.03878 | 0.8126 | ARHGAP22 (margin: ARHGAP22) | 6.41 | 1.92e-07 | 9.88e-04 |
|  | 3 | 57541583 | 57542027 | PDFSTATSENSEMBLUCSC | 445 | 11 | 29184 | island | 0.01697 | 0.5606 | PDE12 (margin: RNU6-483P; PDE12; DNAH12) | 6.32 | 2.84e-06 | 1.46e-02 |
|  | 21 | 39493025 | 39493629 | PDFSTATSENSEMBLUCSC | 605 | 11 | 26504 | tss | 0.03988 | 0.7984 | DSCR8; DSCR4 (margin: DSCR8; DSCR4) | 6.29 | 2.84e-06 | 1.46e-02 |
|  | 5 | 94982161 | 94982715 | PDFSTATSENSEMBLUCSC | 555 | 11 | 32907 | island | 0.02558 | 0.6627 | RFESD (margin: RFESD; SPATA9) | 6.18 | 2.84e-06 | 1.46e-02 |
|  | X | 134186198 | 134186640 | PDFSTATSENSEMBLUCSC | 443 | 11 | 42048 | island | 0.01875 | 0.5412 | FAM127B (margin: FAM127B) | 6.15 | 2.84e-06 | 1.46e-02 |
|  | 6 | 32116538 | 32117725 | PDFSTATSENSEMBLUCSC | 1188 | 27 | 33995 | gene;island | 0.04216 | 0.8349 | PRRT1 (margin: PPT2; PPT2-EGFL8; PRRT1) | 6.10 | 7.75e-10 | 3.98e-06 |
|  | 3 | 130745442 | 130745959 | PDFSTATSENSEMBLUCSC | 518 | 13 | 28549 | tss;island | 0.02093 | 0.5923 | NEK11; ASTE1 (margin: ATP2C1; NEK11; ASTE1) | 6.07 | 1.92e-07 | 9.88e-04 |
|  | 20 | 61051317 | 61052060 | PDFSTATSENSEMBLUCSC | 744 | 18 | 25833 | tss;island | 0.07097 | 0.8311 | RP13-379O24.3; GATA5 (margin: RP13-379O24.3; GATA5) | 6.04 | 2.20e-10 | 1.13e-06 |
|  | 13 | 111267667 | 111268031 | PDFSTATSENSEMBLUCSC | 365 | 11 | 11851 | island | 0.68442 | 0.0378 | CARKD (margin: CARKD) | 6.04 | 2.84e-06 | 1.46e-02 |
|  | 18 | 22006316 | 22006735 | PDFSTATSENSEMBLUCSC | 420 | 11 | 19403 | island | 0.02611 | 0.6046 | IMPACT (margin: IMPACT; Y\_RNA; RP11-178F10.1) | 6.01 | 2.84e-06 | 1.46e-02 |
|  | 3 | 44770851 | 44771316 | PDFSTATSENSEMBLUCSC | 466 | 11 | 28998 | island | 0.54117 | 0.0199 | ZNF501 (margin: ZNF502; ZNF501; KIAA1143) | 6.00 | 2.84e-06 | 1.46e-02 |
|  | X | 77150966 | 77151316 | PDFSTATSENSEMBLUCSC | 351 | 11 | 41884 | island | 0.02388 | 0.5842 | MAGT1 (margin: RN7SL460P; COX7B; MAGT1) | 5.99 | 2.84e-06 | 1.46e-02 |
|  | 19 | 12146404 | 12147014 | PDFSTATSENSEMBLUCSC | 611 | 13 | 21553 | island | 0.02165 | 0.5203 | CTD-2006C1.2; ZNF433; CTD-2006C1.10 (margin: CTD-2006C1.2; ZNF433; CTD-2006C1.10; RNA5SP465; ZNF878) | 5.98 | 1.92e-07 | 9.88e-04 |
|  | 6 | 18122938 | 18123232 | PDFSTATSENSEMBLUCSC | 295 | 11 | 34594 | tss;island | 0.03106 | 0.6695 | NHLRC1 (margin: NHLRC1; TPMT) | 5.98 | 2.84e-06 | 1.46e-02 |
|  | 22 | 22901569 | 22902237 | PDFSTATSENSEMBLUCSC | 669 | 11 | 26963 | tss;island | 0.03729 | 0.7707 | LL22NC03-63E9.3; PRAME (margin: LL22NC03-63E9.3; PRAME) | 5.97 | 2.84e-06 | 1.46e-02 |
|  | 12 | 54331892 | 54332695 | PDFSTATSENSEMBLUCSC | 804 | 12 | 10451 | island | 0.01790 | 0.5469 | HOXC13; HOXC-AS5 (margin: HOXC13; HOXC-AS5) | 5.96 | 1.48e-06 | 7.60e-03 |
|  | X | 153059760 | 153060546 | PDFSTATSENSEMBLUCSC | 787 | 11 | 41587 | tss;island | 0.01792 | 0.5587 | SSR4; IDH3G (margin: SRPK3; SSR4; IDH3G; PDZD4) | 5.92 | 2.84e-06 | 1.46e-02 |
|  | 15 | 45670865 | 45671347 | PDFSTATSENSEMBLUCSC | 483 | 13 | 13304 | gene;tss;island | 0.91080 | 0.0655 | GATM (margin: GATM) | 5.79 | 8.65e-06 | 4.45e-02 |
|  | X | 153775262 | 153776107 | PDFSTATSENSEMBLUCSC | 846 | 16 | 41164 | gene;tss;island | 0.02333 | 0.5901 | IKBKG; G6PD (margin: IKBKG; G6PD) | 5.77 | 3.33e-09 | 1.71e-05 |
|  | 2 | 24232603 | 24232974 | PDFSTATSENSEMBLUCSC | 372 | 11 | 24087 | island | 0.25470 | 0.9404 | MFSD2B (margin: UBXN2A; MFSD2B) | 5.75 | 2.84e-06 | 1.46e-02 |
|  | X | 47518016 | 47518849 | PDFSTATSENSEMBLUCSC | 834 | 17 | 40982 | gene;island | 0.02432 | 0.5207 | RP1-212G6.7; UXT (margin: RP1-212G6.7; ELK1; UXT) | 5.74 | 3.43e-09 | 1.76e-05 |
|  | 20 | 32307885 | 32308529 | PDFSTATSENSEMBLUCSC | 645 | 11 | 26062 | island | 0.02402 | 0.6168 | PXMP4 (margin: ZNF341; PXMP4; RP4-553F4.2) | 5.71 | 2.84e-06 | 1.46e-02 |
|  | 1 | 68515788 | 68516713 | PDFSTATSENSEMBLUCSC | 926 | 17 | 3362 | island | 0.05192 | 0.7287 | GNG12-AS1; DIRAS3; ARL5AP3 (margin: GNG12-AS1; RP4-609E1.2; AL157407.1; DIRAS3; ARL5AP3) | 5.70 | 8.57e-10 | 4.40e-06 |
|  | 1 | 68515788 | 68516453 | PDFSTATSENSEMBLUCSC | 666 | 11 | 573 | gene | 0.05192 | 0.6677 | GNG12-AS1; DIRAS3; ARL5AP3 (margin: GNG12-AS1; RP4-609E1.2; AL157407.1; DIRAS3; ARL5AP3) | 5.70 | 2.84e-06 | 1.46e-02 |
|  | 3 | 37033625 | 37034787 | PDFSTATSENSEMBLUCSC | 1163 | 22 | 27674 | gene | 0.39558 | 0.0145 | MLH1; EPM2AIP1 (margin: MLH1; RP11-129K12.4; RP11-129K12.3; EPM2AIP1) | 5.52 | 6.65e-12 | 3.42e-08 |
|  | 2 | 198364525 | 198365150 | PDFSTATSENSEMBLUCSC | 626 | 16 | 22931 | gene;tss;island | 0.02383 | 0.4748 | HSPE1; MOB4; HSPD1 (margin: HSPE1; MOB4; HSPD1) | 5.45 | 3.33e-09 | 1.71e-05 |
|  | 17 | 5094881 | 5095455 | PDFSTATSENSEMBLUCSC | 575 | 11 | 17858 | island | 0.02901 | 0.5372 | RP11-333E1.1; ZNF594 (margin: RP11-333E1.1; ZNF594) | 5.36 | 2.84e-06 | 1.46e-02 |
|  | 5 | 114937535 | 114938459 | PDFSTATSENSEMBLUCSC | 925 | 11 | 31664 | gene;island | 0.12126 | 0.8848 | AC010226.4; TICAM2; TMED7-TICAM2 (margin: AC010226.4; TICAM2; TMED7-TICAM2; TMED7) | 5.35 | 2.84e-06 | 1.46e-02 |
|  | 18 | 71815073 | 71815372 | PDFSTATSENSEMBLUCSC | 300 | 11 | 19285 | tss;island | 0.01393 | 0.3799 | TIMM21; FBXO15 (margin: TIMM21; FBXO15) | 5.34 | 2.84e-06 | 1.46e-02 |
|  | 3 | 44753865 | 44754587 | PDFSTATSENSEMBLUCSC | 723 | 12 | 28997 | island | 0.55584 | 0.0353 | ZNF502; SOCS5P3 (margin: ZNF502; SOCS5P3) | 5.30 | 7.40e-07 | 3.80e-03 |
|  | 15 | 98503714 | 98503952 | PDFSTATSENSEMBLUCSC | 239 | 11 | 14465 | island | 0.02180 | 0.4660 | ARRDC4 (margin: ARRDC4) | 5.29 | 2.84e-06 | 1.46e-02 |
|  | 1 | 74663583 | 74664160 | PDFSTATSENSEMBLUCSC | 578 | 11 | 1692 | tss;island | 0.02126 | 0.5129 | FPGT; FPGT-TNNI3K; TNNI3K; LRRIQ3 (margin: FPGT; FPGT-TNNI3K; TNNI3K; LRRIQ3) | 5.25 | 2.84e-06 | 1.46e-02 |
|  | 8 | 1764878 | 1765477 | PDFSTATSENSEMBLUCSC | 600 | 12 | 39974 | island | 0.03227 | 0.5346 | MIR596 (margin: MIR596; ARHGEF10) | 5.16 | 7.40e-07 | 3.80e-03 |
|  | 12 | 54410251 | 54410632 | PDFSTATSENSEMBLUCSC | 382 | 12 | 9729 | tss | 0.02116 | 0.3566 | HOXC5; HOXC6; HOXC4 (margin: HOXC5; HOXC6; HOXC8; HOXC4; AC012531.25) | 5.04 | 5.18e-06 | 2.66e-02 |
|  | 22 | 45680498 | 45681036 | PDFSTATSENSEMBLUCSC | 539 | 12 | 27531 | island | 0.87809 | 0.1717 | UPK3A (margin: CTA-268H5.9; UPK3A) | 4.98 | 7.40e-07 | 3.80e-03 |
|  | 12 | 54410251 | 54410727 | PDFSTATSENSEMBLUCSC | 477 | 17 | 10467 | island | 0.01517 | 0.3018 | HOXC5; HOXC6; HOXC4 (margin: HOXC5; HOXC6; HOXC8; HOXC4; AC012531.25) | 4.98 | 6.00e-09 | 3.08e-05 |
|  | 11 | 47270041 | 47270669 | PDFSTATSENSEMBLUCSC | 629 | 14 | 6586 | gene;tss;island | 0.02928 | 0.4591 | NR1H3; ACP2 (margin: DDB2; NR1H3; ACP2) | 4.96 | 4.99e-08 | 2.56e-04 |
|  | 1 | 111506233 | 111507160 | PDFSTATSENSEMBLUCSC | 928 | 17 | 3576 | island | 0.01602 | 0.3172 | LRIF1 (margin: RP11-96K19.2; LRIF1) | 4.94 | 8.57e-10 | 4.40e-06 |
|  | 19 | 34174665 | 34175554 | PDFSTATSENSEMBLUCSC | 890 | 21 | 19939 | gene;island | 0.08304 | 0.7398 | CHST8 (margin: CHST8) | 4.92 | 1.29e-06 | 6.63e-03 |
|  | 20 | 61148178 | 61149009 | PDFSTATSENSEMBLUCSC | 832 | 12 | 25449 | gene;island | 0.14751 | 0.8280 | C20orf166; C20orf166-AS1 (margin: C20orf166; MIR1-1; C20orf166-AS1) | 4.90 | 7.40e-07 | 3.80e-03 |
|  | 5 | 80597143 | 80597828 | PDFSTATSENSEMBLUCSC | 686 | 13 | 31610 | gene;island | 0.44632 | 0.0217 | ZCCHC9; CTC-281B15.1 (margin: ZCCHC9; CTC-281B15.1) | 4.90 | 1.92e-07 | 9.88e-04 |
|  | 16 | 88717374 | 88717989 | PDFSTATSENSEMBLUCSC | 616 | 13 | 16176 | island | 0.78964 | 0.0948 | CYBA; MVD (margin: IL17C; SNAI3-AS1; CYBA; MVD) | 4.83 | 3.85e-07 | 1.98e-03 |
|  | 11 | 2019436 | 2020560 | PDFSTATSENSEMBLUCSC | 1125 | 36 | 7035 | tss;island | 0.02256 | 0.4613 | H19 (margin: AC051649.6; MRPL23-AS1; H19) | 4.81 | 9.15e-13 | 4.70e-09 |
|  | 11 | 2721207 | 2722713 | PDFSTATSENSEMBLUCSC | 1507 | 41 | 6448 | gene;island | 0.55738 | 0.0426 | KCNQ1; KCNQ1OT1 (margin: KCNQ1; KCNQ1OT1) | 4.79 | 4.09e-13 | 2.10e-09 |
|  | 7 | 27195918 | 27196555 | PDFSTATSENSEMBLUCSC | 638 | 11 | 38289 | island | 0.02090 | 0.3471 | HOXA-AS3; RP1-170O19.21; HOXA7 (margin: HOXA-AS3; RP1-170O19.21; HOXA-AS4; HOXA3; HOXA6; HOXA7; HOXA9; RP1-170O19.20) | 4.75 | 2.84e-06 | 1.46e-02 |
|  | 11 | 2721243 | 2722713 | PDFSTATSENSEMBLUCSC | 1471 | 40 | 7057 | tss | 0.55688 | 0.0454 | KCNQ1; KCNQ1OT1 (margin: KCNQ1; KCNQ1OT1) | 4.71 | 1.16e-12 | 5.98e-09 |
|  | 1 | 111506577 | 111507160 | PDFSTATSENSEMBLUCSC | 584 | 11 | 1817 | tss | 0.01602 | 0.2892 | LRIF1 (margin: RP11-96K19.2; LRIF1) | 4.66 | 2.84e-06 | 1.46e-02 |
|  | 1 | 233086060 | 233086622 | PDFSTATSENSEMBLUCSC | 563 | 12 | 4318 | island | 0.38720 | 0.0259 | NTPCR (margin: NTPCR) | 4.62 | 7.40e-07 | 3.80e-03 |
|  | 16 | 88717464 | 88717989 | PDFSTATSENSEMBLUCSC | 526 | 11 | 15380 | tss | 0.67959 | 0.1053 | CYBA; MVD (margin: IL17C; SNAI3-AS1; CYBA; MVD) | 4.60 | 5.67e-06 | 2.91e-02 |
|  | 3 | 49066699 | 49067151 | PDFSTATSENSEMBLUCSC | 453 | 11 | 29063 | island | 0.02423 | 0.3306 | RP13-131K19.6; IMPDH2; QRICH1 (margin: NDUFAF3; RP13-131K19.6; DALRD3; MIR425; MIR191; IMPDH2; QRICH1) | 4.54 | 2.84e-06 | 1.46e-02 |
|  | 17 | 46688182 | 46688845 | PDFSTATSENSEMBLUCSC | 664 | 13 | 18455 | island | 0.01769 | 0.2495 | HOXB7; HOXB8 (margin: HOXB-AS3; HOXB3; HOXB6; HOXB7; HOXB8; HOXB9) | 4.51 | 1.92e-07 | 9.88e-04 |
|  | 6 | 111580120 | 111580939 | PDFSTATSENSEMBLUCSC | 820 | 15 | 36357 | island | 0.42121 | 0.0237 | KIAA1919; RP11-428F8.2 (margin: KIAA1919; RP11-428F8.2) | 4.47 | 6.55e-06 | 3.37e-02 |
|  | 20 | 57425979 | 57428032 | PDFSTATSENSEMBLUCSC | 2054 | 65 | 25433 | gene;tss;island | 0.49326 | 0.0377 | GNAS; GNAS-AS1 (margin: GNAS; GNAS-AS1; RP1-309F20.3) | 4.44 | 6.13e-17 | 3.15e-13 |
|  | 11 | 86383182 | 86383585 | PDFSTATSENSEMBLUCSC | 404 | 12 | 6835 | gene | 0.05540 | 0.5635 | ME3 (margin: ME3) | 4.44 | 7.40e-07 | 3.80e-03 |
|  | 5 | 135415693 | 135416613 | PDFSTATSENSEMBLUCSC | 921 | 16 | 33084 | island | 0.05114 | 0.5330 | VTRNA2-1 (margin: VTRNA2-1) | 4.42 | 3.33e-09 | 1.71e-05 |
|  | 3 | 181428046 | 181428697 | PDFSTATSENSEMBLUCSC | 652 | 15 | 27998 | gene;island | 0.43117 | 0.0413 | SOX2-OT; SOX2 (margin: SOX2-OT; SOX2) | 4.37 | 1.29e-08 | 6.63e-05 |
|  | 1 | 26233332 | 26233709 | PDFSTATSENSEMBLUCSC | 378 | 11 | 1376 | tss;island | 0.04786 | 0.4992 | STMN1; MIR3917 (margin: AL033528.1; STMN1; MIR3917) | 4.34 | 2.84e-06 | 1.46e-02 |
|  | 8 | 11560299 | 11560851 | PDFSTATSENSEMBLUCSC | 553 | 12 | 39489 | tss;island | 0.73098 | 0.1674 | GATA4 (margin: GATA4) | 4.31 | 7.40e-07 | 3.80e-03 |
|  | 3 | 181428242 | 181428697 | PDFSTATSENSEMBLUCSC | 456 | 14 | 28696 | tss | 0.41932 | 0.0412 | SOX2-OT; SOX2 (margin: SOX2-OT; SOX2) | 4.31 | 4.99e-08 | 2.56e-04 |
|  | 19 | 34174665 | 34175419 | PDFSTATSENSEMBLUCSC | 755 | 19 | 20652 | tss | 0.08304 | 0.7398 | CHST8 (margin: CHST8) | 4.31 | 9.13e-06 | 4.69e-02 |
|  | 5 | 140810051 | 140811312 | PDFSTATSENSEMBLUCSC | 1262 | 16 | 31779 | gene;island | 0.34269 | 0.8951 | PCDHGA1; PCDHGA2; PCDHGA3; PCDHGB1; PCDHGA4; PCDHGB2; PCDHGA5; PCDHGB3; PCDHGA6; PCDHGA7; PCDHGB4; PCDHGA8; PCDHGA9; PCDHGB6; PCDHGA10; PCDHGB7; PCDHGA11; PCDHGB8P; PCDHGA12 (margin: PCDHGA1; PCDHGA2; PCDHGA3; PCDHGB1; PCDHGA4; PCDHGB2; PCDHGA5; PCDHGB3; PCDHGA6; PCDHGA7; PCDHGB4; PCDHGA8; PCDHGA9; PCDHGB6; PCDHGA10; PCDHGB7; PCDHGA11; PCDHGB8P; PCDHGA12) | 4.28 | 1.33e-08 | 6.84e-05 |
|  | 6 | 122931154 | 122931702 | PDFSTATSENSEMBLUCSC | 549 | 11 | 34398 | gene;island | 0.06450 | 0.5717 | PKIB (margin: PKIB) | 4.28 | 2.84e-06 | 1.46e-02 |
|  | 3 | 44626029 | 44626860 | PDFSTATSENSEMBLUCSC | 832 | 11 | 28993 | island | 0.31663 | 0.0327 | ZKSCAN7; ZNF660; ZNF197; RP11-944L7.4 (margin: ZKSCAN7; ZNF660; ZNF197; RP11-944L7.4; MPRIPP1) | 4.27 | 5.67e-06 | 2.91e-02 |
|  | 6 | 32133929 | 32135396 | PDFSTATSENSEMBLUCSC | 1468 | 44 | 34002 | gene;island | 0.30898 | 0.8807 | PPT2; PPT2-EGFL8; EGFL8; AGPAT1 (margin: PPT2; PPT2-EGFL8; EGFL8; RNF5; PRRT1; AGPAT1) | 4.26 | 1.57e-13 | 8.07e-10 |
|  | 1 | 19615303 | 19615702 | PDFSTATSENSEMBLUCSC | 400 | 11 | 1298 | tss;island | 0.11750 | 0.8176 | AKR7A3 (margin: RP4-657E11.10; AKR7A3) | 4.26 | 2.84e-06 | 1.46e-02 |
|  | 19 | 11784246 | 11785337 | PDFSTATSENSEMBLUCSC | 1092 | 15 | 21544 | island | 0.03751 | 0.5119 | ZNF833P (margin: ZNF833P; CTC-499B15.4; HNRNPA1P10) | 4.24 | 2.45e-07 | 1.26e-03 |
|  | 1 | 67217673 | 67218275 | PDFSTATSENSEMBLUCSC | 603 | 12 | 3354 | island | 0.02380 | 0.3589 | TCTEX1D1 (margin: SGIP1; TCTEX1D1) | 4.16 | 7.40e-07 | 3.80e-03 |
|  | 7 | 27154262 | 27155548 | PDFSTATSENSEMBLUCSC | 1287 | 18 | 36868 | gene;island | 0.70517 | 0.0809 | HOXA-AS2; HOXA3 (margin: HOXA-AS2; HOXA2; HOXA3) | 4.14 | 9.92e-09 | 5.10e-05 |
|  | 10 | 135050619 | 135051351 | PDFSTATSENSEMBLUCSC | 733 | 25 | 5438 | tss | 0.32901 | 0.0283 | VENTX (margin: KNDC1; UTF1; VENTX; MIR202) | 4.14 | 1.90e-13 | 9.76e-10 |
|  | 8 | 97657072 | 97657833 | PDFSTATSENSEMBLUCSC | 762 | 14 | 40410 | island | 0.07953 | 0.5720 | CPQ (margin: CPQ) | 4.13 | 4.99e-08 | 2.56e-04 |
|  | 6 | 32063394 | 32064258 | PDFSTATSENSEMBLUCSC | 865 | 28 | 33984 | gene;island | 0.37747 | 0.9253 | TNXB; ATF6B (margin: TNXB; ATF6B) | 4.12 | 7.11e-09 | 3.65e-05 |
|  | 10 | 135050619 | 135051581 | PDFSTATSENSEMBLUCSC | 963 | 28 | 6330 | island | 0.33540 | 0.0291 | VENTX (margin: KNDC1; UTF1; VENTX; MIR202) | 4.09 | 1.03e-09 | 5.31e-06 |
|  | 7 | 150497065 | 150497760 | PDFSTATSENSEMBLUCSC | 696 | 11 | 37214 | gene;tss;island | 0.30002 | 0.8561 | TMEM176A; TMEM176B (margin: RP5-1051J4.4; TMEM176A; TMEM176B) | 4.06 | 2.84e-06 | 1.46e-02 |
|  | 12 | 51566379 | 51567112 | PDFSTATSENSEMBLUCSC | 734 | 11 | 10396 | island | 0.44877 | 0.0410 | TFCP2 (margin: RNU6-199P; TFCP2) | 4.02 | 2.84e-06 | 1.46e-02 |
|  | 6 | 31165567 | 31166174 | PDFSTATSENSEMBLUCSC | 608 | 20 | 33800 | gene | 0.02592 | 0.2824 | HCG27; XXbac-BPG299F13.17; XXbac-BPG299F13.14 (margin: HCG27; XXbac-BPG299F13.17; XXbac-BPG299F13.14) | 3.89 | 1.69e-07 | 8.68e-04 |
|  | 6 | 31165290 | 31166174 | PDFSTATSENSEMBLUCSC | 885 | 29 | 35694 | island | 0.02763 | 0.2758 | HCG27; XXbac-BPG299F13.17; XXbac-BPG299F13.14 (margin: HCG27; XXbac-BPG299F13.17; XXbac-BPG299F13.14) | 3.86 | 1.43e-11 | 7.33e-08 |
|  | X | 46404528 | 46405125 | PDFSTATSENSEMBLUCSC | 598 | 14 | 41271 | tss;island | 0.08432 | 0.5946 | ZNF674-AS1; ZNF674 (margin: ZNF674-AS1; ZNF674) | 3.80 | 4.99e-08 | 2.56e-04 |
|  | 2 | 27805536 | 27806117 | PDFSTATSENSEMBLUCSC | 582 | 11 | 24151 | island | 0.92048 | 0.4925 | C2orf16; ZNF512; RP11-158I13.2; AC074091.1 (margin: C2orf16; ZNF512; RP11-158I13.2; AC074091.1) | 3.66 | 5.67e-06 | 2.91e-02 |
|  | 6 | 30923241 | 30924004 | PDFSTATSENSEMBLUCSC | 764 | 17 | 35684 | island | 0.35674 | 0.0450 | DPCR1; HCG21 (margin: DPCR1; HCG21) | 3.65 | 5.86e-07 | 3.01e-03 |
|  | 7 | 27154262 | 27155039 | PDFSTATSENSEMBLUCSC | 778 | 14 | 37425 | tss | 0.64307 | 0.0701 | HOXA-AS2; HOXA3 (margin: HOXA-AS2; HOXA2; HOXA3) | 3.65 | 1.50e-06 | 7.69e-03 |
|  | X | 48931388 | 48932122 | PDFSTATSENSEMBLUCSC | 735 | 12 | 41781 | island | 0.20282 | 0.0244 | PRAF2; WDR45 (margin: CCDC120; RNU4-52P; PRAF2; WDR45) | 3.59 | 1.48e-06 | 7.60e-03 |
|  | 3 | 48632484 | 48633144 | PDFSTATSENSEMBLUCSC | 661 | 11 | 29047 | island | 0.38028 | 0.8459 | COL7A1 (margin: COL7A1; UQCRC1) | 3.50 | 5.67e-06 | 2.91e-02 |
|  | 10 | 135049991 | 135050355 | PDFSTATSENSEMBLUCSC | 365 | 13 | 5437 | tss;island | 0.33678 | 0.0307 | VENTX (margin: KNDC1; UTF1; VENTX; MIR202) | 3.48 | 1.35e-06 | 6.92e-03 |
|  | 10 | 105212056 | 105212813 | PDFSTATSENSEMBLUCSC | 758 | 12 | 6084 | island | 0.38751 | 0.8423 | RP11-225H22.4; CALHM2; CALHM1 (margin: PDCD11; RP11-225H22.4; CALHM2; CALHM1) | 3.40 | 7.40e-07 | 3.80e-03 |
|  | 11 | 2889809 | 2890725 | PDFSTATSENSEMBLUCSC | 917 | 32 | 7058 | tss | 0.34222 | 0.8662 | KCNQ1DN (margin: KCNQ1DN; KCNQ1-AS1) | 3.35 | 5.08e-08 | 2.61e-04 |
|  | 6 | 28601269 | 28601519 | PDFSTATSENSEMBLUCSC | 251 | 14 | 35529 | island | 0.89665 | 0.4589 | RP11-373N24.2 (margin: RP11-373N24.2) | 3.31 | 5.98e-07 | 3.07e-03 |
|  | 11 | 2889602 | 2890725 | PDFSTATSENSEMBLUCSC | 1124 | 35 | 7955 | island | 0.33717 | 0.8580 | KCNQ1DN (margin: KCNQ1DN; KCNQ1-AS1) | 3.26 | 7.38e-08 | 3.79e-04 |
|  | 6 | 29973988 | 29975427 | PDFSTATSENSEMBLUCSC | 1440 | 47 | 33643 | gene;island | 0.16563 | 0.6928 | HLA-J; ZNRD1-AS1; HCG4P3 (margin: HLA-J; MCCD1P2; ZNRD1-AS1; HCG4P3) | 3.22 | 8.15e-23 | 4.19e-19 |
|  | 6 | 30881112 | 30881842 | PDFSTATSENSEMBLUCSC | 731 | 23 | 33772 | gene | 0.07611 | 0.7018 | GTF2H4; VARS2 (margin: RN7SL175P; GTF2H4; VARS2) | 3.19 | 1.18e-06 | 6.04e-03 |
|  | 3 | 50388115 | 50388670 | PDFSTATSENSEMBLUCSC | 556 | 14 | 27766 | gene;island | 0.18697 | 0.0275 | CYB561D2; XXcos-LUCA11.5; NPRL2 (margin: ZMYND10-AS1; CYB561D2; XXcos-LUCA11.5; RASSF1; ZMYND10; NPRL2; TMEM115; CACNA2D2) | 3.08 | 4.99e-08 | 2.56e-04 |
|  | X | 48754982 | 48755797 | PDFSTATSENSEMBLUCSC | 816 | 21 | 40988 | gene;tss;island | 0.24712 | 0.0275 | PQBP1; TIMM17B (margin: PQBP1; TIMM17B; SLC35A2) | 3.06 | 8.47e-08 | 4.35e-04 |
|  | 10 | 105212236 | 105212813 | PDFSTATSENSEMBLUCSC | 578 | 11 | 5321 | tss | 0.46931 | 0.8449 | RP11-225H22.4; CALHM2; CALHM1 (margin: PDCD11; RP11-225H22.4; CALHM2; CALHM1) | 3.03 | 2.84e-06 | 1.46e-02 |
|  | 10 | 106399877 | 106400880 | PDFSTATSENSEMBLUCSC | 1004 | 11 | 6101 | island | 0.05418 | 0.2667 | SORCS3 (margin: SORCS3) | 2.96 | 5.67e-06 | 2.91e-02 |
|  | 2 | 27851409 | 27852231 | PDFSTATSENSEMBLUCSC | 823 | 16 | 22595 | gene;tss | 0.42328 | 0.0633 | ZNF512; RP11-158I13.2; GPN1; CCDC121 (margin: ZNF512; RP11-158I13.2; GPN1; CCDC121) | 2.92 | 3.04e-06 | 1.56e-02 |
|  | 15 | 79383385 | 79383980 | PDFSTATSENSEMBLUCSC | 596 | 14 | 13814 | tss;island | 0.02511 | 0.1878 | RASGRF1 (margin: RASGRF1) | 2.89 | 6.93e-06 | 3.56e-02 |
|  | 6 | 30139421 | 30140417 | PDFSTATSENSEMBLUCSC | 997 | 26 | 33676 | gene;island | 0.33140 | 0.7849 | TRIM15 (margin: TRIM15; TRIM10; TRIM26) | 2.88 | 4.73e-11 | 2.43e-07 |
|  | 3 | 49026934 | 49027409 | PDFSTATSENSEMBLUCSC | 476 | 13 | 29059 | island | 0.53558 | 0.9015 | P4HTM; RP13-131K19.2 (margin: ARIH2; P4HTM; RP13-131K19.1; RP13-131K19.2) | 2.81 | 8.65e-06 | 4.45e-02 |
|  | 17 | 33775295 | 33776116 | PDFSTATSENSEMBLUCSC | 822 | 12 | 18181 | island | 0.45261 | 0.8582 | SLFN13 (margin: SLFN13; RP11-1094M14.7) | 2.78 | 7.40e-07 | 3.80e-03 |
|  | 6 | 32064491 | 32065211 | PDFSTATSENSEMBLUCSC | 721 | 29 | 33985 | gene;island | 0.43415 | 0.8210 | TNXB; ATF6B (margin: TNXB; ATF6B) | 2.75 | 1.79e-07 | 9.18e-04 |
|  | 1 | 2344979 | 2345475 | PDFSTATSENSEMBLUCSC | 497 | 11 | 1170 | tss;island | 0.55131 | 0.1667 | PEX10 (margin: RER1; PLCH2; PEX10) | 2.52 | 2.84e-06 | 1.46e-02 |
|  | 20 | 36148133 | 36149455 | PDFSTATSENSEMBLUCSC | 1323 | 41 | 25378 | gene;tss;island | 0.93272 | 0.6705 | NNAT; BLCAP (margin: NNAT; PPIAP3; RP11-425M5.5; BLCAP) | 2.52 | 3.32e-07 | 1.71e-03 |
|  | 11 | 44331905 | 44333192 | PDFSTATSENSEMBLUCSC | 1288 | 39 | 7240 | tss;island | 0.45237 | 0.1245 | ALX4 (margin: ALX4) | 2.34 | 1.44e-07 | 7.40e-04 |
|  | 19 | 38182773 | 38183456 | PDFSTATSENSEMBLUCSC | 684 | 11 | 21925 | island | 0.16685 | 0.0468 | ZFP30; ZNF781; CTD-2528L19.4; CTD-2528L19.3 (margin: ZFP30; ZNF781; CTD-2528L19.4; CTD-2528L19.3; ZNF607) | 2.28 | 2.84e-06 | 1.46e-02 |
|  | 6 | 33160067 | 33160604 | PDFSTATSENSEMBLUCSC | 538 | 15 | 35906 | island | 0.12477 | 0.3872 | COL11A2; RXRB (margin: RNY4P10; SLC39A7; HSD17B8; COL11A2; RXRB) | 2.17 | 8.82e-06 | 4.53e-02 |
|  | 3 | 37033625 | 37035399 | PDFSTATSENSEMBLUCSC | 1775 | 43 | 28178 | tss;island | 0.15867 | 0.0196 | MLH1; EPM2AIP1 (margin: MLH1; RP11-129K12.4; RP11-129K12.3; EPM2AIP1) | 2.17 | 1.87e-09 | 9.60e-06 |
|  | 1 | 25257505 | 25258264 | PDFSTATSENSEMBLUCSC | 760 | 20 | 1361 | tss | 0.35193 | 0.0887 | RUNX3 (margin: RUNX3) | 2.05 | 8.34e-08 | 4.29e-04 |
|  | 1 | 25257505 | 25258332 | PDFSTATSENSEMBLUCSC | 828 | 21 | 330 | gene;island | 0.33984 | 0.0994 | RUNX3 (margin: RUNX3) | 1.98 | 3.44e-08 | 1.77e-04 |
|  | 6 | 30131001 | 30132226 | PDFSTATSENSEMBLUCSC | 1226 | 24 | 33673 | gene | 0.36161 | 0.6317 | TRIM15 (margin: TRIM15; TRIM10) | 1.62 | 4.20e-06 | 2.16e-02 |
